# Supplementary material for: Identification of photoperiod-induced specific miRNAs in the adrenal glands of Sunite sheep (Ovis aries)
Source: Front Vet Sci. 2022 Jul 22;9:888207. doi: 10.3389/fvets.2022.888207 (PMC9354845; doi:10.3389/fvets.2022.888207)
Supplement: Supplementary file 1 [file Data_Sheet_1.ZIP › Supplementary file/miRDeep2 file.zip!/miRDeep2 file/Novel_Structure/LP42A1/Novel_139.pdf]

Provisional ID	:	NC_019484.2_36129
Score total	:	64.1
Score for star read(s)	:	3.9
Score for read counts	:	56.7
Score for mfe	:	1.9
Score for randfold	:	1.6
Score for cons. seed	:	
Total read count	:	123
Mature read count	:	122
Loop read count	:	0
Star read count	:	1

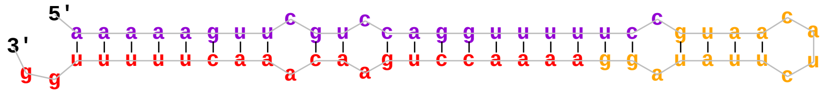

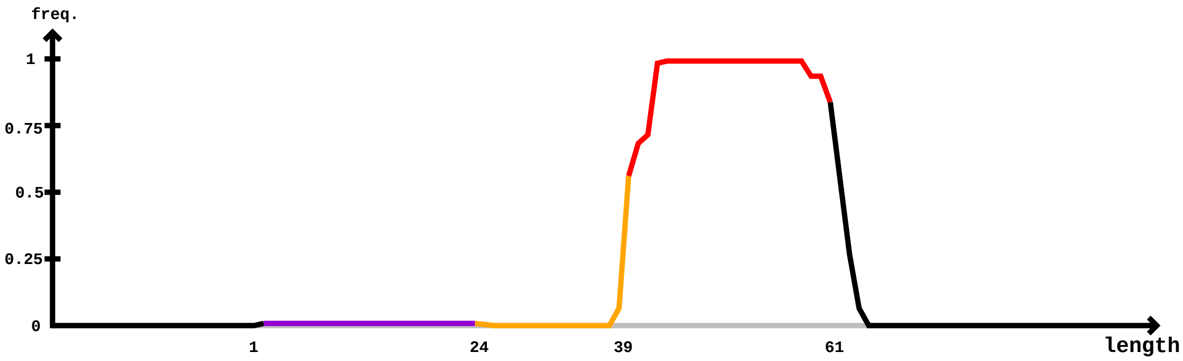

Star Mature

```
5'- uaagcaacuaaugguuggccaaaaaguucguccagguuuuuccguaacaucuuauaggaaaaccugaacaaacuuuuuggccaacccaauauuuuagcauuauuuucuccucu  -3'  obs
    uaagcaacuaaugguuggccaaaaaguucguccagguuuuuccguaacaucuuauaggaaaaccugaacaaacuuuuuggccaacccaauauuuuagcauuauuuucuccucu       exp
    ............((((((((((((((((.((.((((((((((.((((....)))).)))))))))).)).)))))))))))))))).........................    reads    mm    sample
    ....................aaaaaguucguccagguuuuucc......................................................................    1    0    seq
    .........................................................gaaaaccugaacaaacuuuG....................................    1    1    seq
    .........................................................gaaaaccugaacaaacuuuuuU..................................    1    1    seq
    .........................................................Aaaaaccugaacaaacuuuuug..................................    5    1    seq
    .........................................................gaaaaccugaacaaacuuuuugA.................................    1    1    seq
    ..........................................................aaaaUcugaacaaacuuuu....................................    4    1    seq
    ..........................................................aaaaccugaacaaacuuuu....................................    1    0    seq
    ..........................................................aaaaccugGacaaacuuuu....................................    1    1    seq
    ..........................................................aaaaccugaacGaacuuuuug..................................    2    1    seq
    ..........................................................aaaaccugaacUaacuuuuug..................................    3    1    seq
    ..........................................................aaaaccugaacaaacuuuuug..................................    1    0    seq
    ..........................................................aaaaccugaacaaacuuuuugg.................................    3    0    seq
    ..........................................................aaaaUcugaacaaacuuuuugg.................................    45    1    seq
    ..........................................................aaaaUcugaacaaacuuuuuggc................................    1    1    seq
    ...........................................................aaaUcugaacaaacuuuuugg.................................    14    1    seq
    ...........................................................aaaAcugaacaaacuuuuuggc................................    1    1    seq
    ............................................................aaUcugaacaaacuuuuuggc................................    4    1    seq
    .............................................................aUcugaacaaacuuuuuggcc...............................    25    1    seq
    .............................................................aUcugaacaaacuuuuuggcca..............................    8    1    seq
    ..............................................................Ucugaacaaacuuuuuggc................................    1    1    seq
```
